# Role of Senescence in Tumorigenesis and Anticancer Therapy

**DOI:** 10.1155/2022/5969536

**Published:** 2022-03-18

**Authors:** Ewelina Stoczynska-Fidelus, Marta Węgierska, Amelia Kierasińska, Damian Ciunowicz, Piotr Rieske

**Affiliations:** ^1^Department of Molecular Biology, Chair of Medical Biology, Medical University of Lodz, Zeligowskiego 7/9 St., 90-752 Lodz, Poland; ^2^Department of Tumor Biology, Chair of Medical Biology, Medical University of Lodz, Zeligowskiego 7/9 St., 90-752 Lodz, Poland

## Abstract

Although the role of senescence in many physiological and pathological processes is becoming more identifiable, many aspects of senescence are still enigmatic. A special attention is paid to the role of this phenomenon in tumor development and therapy. This review mainly deals with a large spectrum of oncological issues, beginning with therapy-induced senescence and ending with oncogene-induced senescence. Moreover, the role of senescence in experimental approaches, such as primary cancer cell culture or reprogramming into stem cells, is also beginning to receive further consideration. Additional focus is made on senescence resulting from mitotic catastrophe processes triggered by events occurring during mitosis and jeopardizing chromosomal stability. It has to be also realized that based on recent findings, the basics of senescent cell property interpretation, such as irreversibility of proliferation blockade, can be undermined. It shows that the definition of senescence probably requires updating. Finally, the role of senescence is lately more understandable in the immune system, especially since senescence can diminish the effectiveness of the chimeric antigen receptor T-cell (CAR-T) therapy. In this review, we summarize the current knowledge regarding all these issues.

## 1. Introduction

According to the vast majority of scientific articles, senescence is defined as an irreversible inhibition of cell proliferation accompanied by processes such as senescence-associated secretion (SAS) and senescence-associated heterochromatin foci (SAHF). Nowadays, senescence analysis is considered an important research field, providing plenty of new, sometimes surprising, results. It is widely accepted that senescence is associated with the inhibition of the cell cycle. Therefore, a link between senescence and suppressors such as p16^INK4a^ or RB is often suggested. However, this statement is an obvious simplification.

Importantly, it is crucial to distinguish temporal inhibition of cell cycle from permanent termination of cell divisions. Senescence should be considered not only as inhibition of cell cycle but also as activation of protein secretion and cell enlargement (growth). More importantly, many research centers emphasize that cancer cells are in fact pro-senescent, contradicting the hypothesis that cancer cells are characterized by an anti-senescent state [1]. According to these scientists, cancer cells are prone to senescence induction, possibly even more than normal cells, and this susceptibility may, but not necessarily, constitute their weak point. The hypothesis is generally based on a widely known phenomenon such as oncogene-induced senescence (OIS). As an example, BRAF or RAS-induced senescence was widely described in the literature [2]. PTEN (loss)-induced cellular senescence (PICS), resulting from lack of PTEN suppressor, was also reported [3, 4]. It is worth making a point of the fact that OIS is much more rapid than senescence resulting from, e.g., shortening of telomeres [5]. It has been found that the inhibition of nuclear factor kappa-light-chain-enhancer of activated B-cell(NF-*κ*B) pathway results in senescence of cancer stem cells [6]. It indicates that the control of cellular processes is much more complex than previously suggested. Showing only one sight of oncogenes and tumor suppressor action does not apply to proliferation *versus* senescence only. Similarly, many oncogenes such as cyclin-dependent kinase (CDK) can stimulate, and suppressors such as RB can block apoptosis.

It is obvious that senescence should not be considered solely as termination of cell divisions but also as activation of a wide range of cellular processes. Clearly, senescent cells enlarge. Moreover, over the last few years, many scientific articles have been focused on the senescence-associated secretory phenotype (SASP). Despite the lack of proliferation, senescent cells remain viable, metabolically active, and may secrete cytokines, growth factors, chemokines, proteases, and many others [7]. Factors secreted by senescent cells may promote migration and invasiveness of cancer cells. Among these factors, IL-6 and IL-8, which act *via* promoting epithelial-to-mesenchymal transition [8], matrix metalloproteinase 3 (MMP3), which by degradation of extracellular matrix facilitates migration of cells, can be enumerated [9, 10]. Additionally, senescent cells are able to secrete vascular endothelial growth factor (VEGF), a growth factor-stimulating angiogenesis [11]. Few publications by Campisi and d'Adda di Fagagna presented the impact of the secretory phenotype of senescent cells on other cells in culture [12].

The secretory phenotype of senescent cells was demonstrated to be associated with the secretion of IL-6 and IL-8, factors that play a crucial role in tumor propagation by stimulating the proliferation of cancer stem cells [13]. Chiou et al. mentioned also that high IL-6 and IL-8 expression correlates with a worse prognosis [14]. By secreting VEGF-A and VEGF-C, factors involved in angiogenesis, senescent cells may be involved in metastasis [13].

Despite the ability of senescent cells to support other neoplastic cells, e.g. secretion of cytokines, the involvement of another intriguing phenomenon seems possible. A classic definition of senescence assumes irreversibility of proliferation inhibition [15]; however, it was based on the analyses of normal cells. In the case of tumor cells, this phenomenon has not been analyzed profoundly enough to be certain that cells characterized by the presence of senescence-associated beta-galactosidase (SA-*β*-Gal) do not reenter the cell cycle. Some studies suggest that senescence may be indeed a reversible process, and therefore, it seems possible that the proliferation abilities of cells with features of senescence can be restored [16]. Although such hypothesis is contradictory to the current senescence definition, a new approach is probably needed since the amount of data showing reversibility of senescence is growing.

Analyses of p21-inducible, p53-null, neoplastic, and preneoplastic *in vitro* models showed that after an initial senescence, p21 causes opposite effects [17]. Senescence can be also disabled in cancer cells by H3K9 active demethylases—the lysine-specific demethylase 1 (LSD1) [18]. In accordance with that, Milanovic et al. developed switchable models of senescence targeting H3K9me3 or TP53 imitating spontaneous escape from the arrested condition. They observed that cells released from senescent cells enhanced Wnt-dependent proliferation potential [19]. Apparently, even senescent cells, thanks to additional mutations, can invert phenotype to proliferative one [20]. It was also shown that cells arrested in oncogene-induced senescence retain the potential to escape senescence by mechanisms that involve, i.e., derepression of hTERT expression [21].

Another intriguing phenomenon to be considered is connected with markers of senescence [22]. The question is whether each senescent cell should be positive for each senescence marker. For example, the lack of SAHF structures in SA-*β*-Gal-positive tumor cells stands in favor of the possibility to restore their proliferation [23]. SAHF structures are very stable foci of heterochromatin generated as a result of RB protein action. As decomposition of these structures is extremely difficult, their lack may support the fact that senescent cancer cells (characterized by the lack of bromodeoxyuridine incorporation following long-term exposure, cell enlargement, high SA-*β*-Gal activity) may restore the ability of mitotic divisions.

Nowadays, when an increasing number of cancer cases are observed, there is a great necessity to discover effective anticancer drugs and therapies [24]. Most of the drugs that are currently available on the market are focused on inducing programmed cell death in cancer cells. In some cases, the apoptosis of cancer cells is too rapid and leads to the development of strong inflammation, which is, in turn, unfavorable for the patient. It can lead to failure of the organ within which the tumor was localized. On the other hand, the inflammation can also lead to excessive proliferation of cancer cells, induction of angiogenesis, or even metastases [25, 26]. Due to the more frequently observed adverse effects of currently used anticancer drugs, researchers are trying to find new alternative approaches to fight cancer. One of the most promising methods is induction of senescence—an irreversible cell cycle arrest [27].

Senescence induction was observed as a side effect of treatment with some regular chemotherapeutics such as cisplatin and doxorubicin. These drugs do not always cause the induction of apoptosis, and they can also lastingly inhibit the proliferative potential of cancer cells [28, 29]. This action initially seems to limit the negative effects of the treatment leading to a similar therapeutic outcome. Unfortunately, senescent cells despite cell cycle arrest still retain metabolic activity able to promote the tumor growth and proliferation of cancer cells (in which senescence has not been induced) located within the tumor. The way to counteract the secretory phenotype is to remove SASP elements, for example, using antibodies against specific factors secreted by senescent cells [29]. The other approach in the prevention of secretory activity of senescent tumor cells is the elimination of these cells *via* senolytic drugs, which may induce apoptosis in senescent cells or initiate inhibition of SASP element release [30]. Considering oncological therapies, senescence does not always have to refer to cancer cells. This phenomenon is also observed in immune cells used to fight and destroy cancer cells, i.e., in CAR-T. It is reported that genetically modified lymphocytes lose their ability to proliferate when being again implemented into the patient's bloodstream. It is possible that the process responsible for the observed tendency is exactly senescence [31].

Cellular senescence does not always have to be induced by drugs used in therapies. Being a natural defense of the cell against carcinogenesis, senescence functions as a physiological mechanism and can be observed when the cell reaches the limit of replication processes or as a result of oncogene activation, called OIS. It lets to avoid the accumulation of mutation within the cell [32]. It has to be emphasized that senescence appears to be a two-faced mechanism, which, on one hand, is favorable and leads to inhibition of tumor growth, but on the other hand it may be an obstacle during cancer treatment. The usage of drugs that induce senescence leads to cell cycle arrest, but how does the human body handle the presence of senescent cells? Senescence is believed to be irreversible; therefore, how is it possible that cancer cells can escape that state and how does it affect cancer therapy? Is it possible to induce senescence only in cancer cells? If not then what would be the result on noncancerous cells? In this article, we would like to discuss the dualistic role of senescence in anticancer treatment and answer the foregoing questions.

## 2. Senescent Cells and Immune System

It is especially important to discuss both the beneficial and adverse roles of cellular senescence. This discussion becomes even more significant as the link between senescence and the immune system has been indicated. It was even suggested that the relative concentrations of factors secreted by senescent tumor cells and the concentrations of other components in the tumor microenvironment determine whether the overall effects of a senescence-inducing secretome are pro-tumoral or antitumoral [33]. In this aspect, the role of senescence in tumor cell growth arrest, together with senescence-associated secretory phenotype, should be considered on two levels. The first one involves the development of cancer, tumor formation, and metastasis, and the second one regards response to anticancer therapies including immunotherapy. During the last few years, many potential secretome-targeted therapeutic strategies to selectively eliminate tumor cells were proposed [34, 35]. On the other hand, mechanisms driving immunoevasion of senescent tumor cells and their link to chemotherapy resistance were clearly shown [36].

On the contrary to senescence as a tumor-protective mechanism in normal cells, in tumors, the senescence phenomenon may be critical in the aspect of escaping the developing tumor from the immune system response with a number of pro-inflammatory factors playing a crucial role. Firstly, the link between senescence and tumor immunity has been shown in an example of centrosome abnormalities. Many publications described centrosome aberration as a hallmark of human cancers contributing to the senescence process [37]. Interestingly, centrosome dysfunction promotes the secretion of multiple inflammatory factors that act as drivers of senescence. More importantly, these aberrations may be responsible for tumor immune escape by triggering an immunosuppressive microenvironment. As a result of centrosome aberrations, the accumulation of double-stranded DNA (dsDNA) is observed, which induces the cGAS-STING pathway; thus, continuous chromosome segregation errors promote cellular invasion and metastasis in a STING-dependent manner [38, 39]. These findings reveal a molecular mechanism of cellular senescence and suggest that modulation of cGAS activity may be a new strategy to treat senescence-associated human diseases [40]. Additionally, in tumor cells with centrosome aberrations, NF-*κ*B canonical and noncanonical signaling is activated, which mediates a so-called extra centrosome-associated secretory phenotype (ECASP). The secretion of IL-8, GDF-15, and ANGPTL4 within ECASP was described [41, 42]. Since the former cytokine is known as the SASP component, within the same mechanism, it recruits Th2 lymphocytes and macrophages to profile the immunosuppressive microenvironment. Moreover, it was shown that centrosome aberrations are linked to decreased tumor neoantigen expression, suppression of major histocompatibility complex (MHC) class I antigen presentation, and decrease in CD8+ T-cell infiltration [37]. All these reactions enable evasion of antitumor immune responses or distant metastasis.

## 3. Therapy-Induced Senescence

Cell cycle arrest is achieved and maintained at the G1 or G2/M stage of the cell cycle, in part due to increased expression of specific cyclin-dependent kinase inhibitors (CDKIs) including p16^INK4a^ (CDKN2A) [43]. A promising approach to the induction of cytostasis in tumor cells is therapy-induced senescence (TIS) [44]. It has been found that cellular ageing can be accelerated, induced by DNA damage, increased oncogenic signaling, and oxidative stress [12].

Much evidence has accumulated to show that ionizing radiation (IR) induces cellular senescence in various types of cancer cells in a dose-dependent manner. In the non-small cell lung cancer (NSCLC) A549 cells, 2 Gy of radiation yielded ∼20% of SA-*β*-Gal-positive cells, whereas 10 Gy generated the SA-*β*-Gal-positive cells in almost 80% of cells. The response to IR is also cell-type-specific. The same dose of IR caused a higher magnitude of senescence in the H460 line of NSCLC, which appeared to be more sensitive to the irradiation [45]. In the case of TP53 wild-type MCF-7 breast cancer cells, a dose of 10 Gy was also sufficient to induce senescence [46]. The pro-senescence activity of IR was also confirmed in other TP53 wild-type cells, including A172 glioblastoma cell line, SKNSH neuroblastoma cells, and HCT-116 colorectal cancer cells [47]. Investigation of the effect of IR on MDA-MB-231 cancer cells with impaired TP53 function showed that the IR did not cause senescence but induced apoptosis, demonstrating that TP53 status plays a role in IR-associated induction of senescence [46].

Importantly, in cells prone to IR-induced senescence, radiation neither suppresses telomerase subunit expression, alters telomerase activity, nor induces telomere shortening. These facts suggest that this type of therapy-induced cell ageing occurs without telomere loss, but with apparent telomere dysfunction (end-to-end fusions) [46]. Senescence independent of telomere shortening has also been observed in irradiated SA-*β*-Gal-positive lung cancer cells [48].

Induced by IR exposition, either apoptosis or senescence in cancer cells may be dependent on the status of securin, a protein involved in replication, DNA repair, and tumor formation [49, 50]. IR-treated wild-type securin colon carcinoma cells were found to undergo apoptosis, whereas in cells lacking securin IR induces senescence [51]. IR-triggered senescence in human securin-deficient breast cancer cells, MDA-MB-231, includes ATM/Chk2, p38 MAPK, AMPK, and NF-*κ*B pathways [52, 53]. It was also shown that, as a result of senescence in response to IR, the activation of two enzymes critical for glycolysis of glyceraldehyde-3-phosphate dehydrogenase and lactate dehydrogenase was observed. Importantly, IR-dependent cell senescence was reduced after inhibition of glycolysis with dichloroacetate [53]. In glioblastoma cells, transition to apoptosis or senescence after IR treatment is determined by a PTEN suppressor. Its high expression directs the irradiated cells towards apoptosis, while its deficiency promotes senescence [54]. In turn, in lung cancer cells, it was found that IR-induced cellular senescence appears to be regulated by miR-34a [45].

Not only IR but also several drugs including, but not limited to, aphidicolin, bleomycin, cisplatin, doxorubicin, etoposide, mitoxantrone, retinol, hydroxyurea, carboplatin, and docetaxel induce senescence of tumor cells [55]. The majority of these drugs mainly work by inducing DNA damage, but they are also agents that generate reactive oxygen species (ROS), DNA polymerase inhibitors, or differentiating factors. Straightening effects of chemotherapeutic agents have been demonstrated in many cancers, including breast, lung, prostate, and colon cancer, as well as regardless of TP53 status [55, 56]. The ability of drugs to induce cellular ageing depends on how the drug works. A comparative analysis using drug concentrations showed that the strongest senescence response was found for DNA-damaging agents, while the weakest effect was observed for drugs targeting microtubules [57]. Yet, it has to be mentioned that cancer cells exposed to chemotherapeutic agents may undergo senescence in addition to either apoptosis or necrosis. The type of cell response is likely related to the magnitude of the stimulus applied: strong action causes cell death, while a weaker stimulus causes senescence. Such a relationship was observed, for example, in prostate neoplastic cells, which underwent apoptosis after the use of 250 nM doxorubicin [58], while the use of 10 times lower concentration led to senescence [59]. Since the process of apoptosis seems to be rapid and usually reflects 24 hours post-treatment, the characteristic hypertrophic morphology of neoplastic cells and SA-*β*-Gal expression takes at least several days (3–7) after treatment to be visible [59]. Importantly, cellular ageing compared with cell death is caused by the use of lower doses of drugs, which is likely to minimize possible side effects of treatment to normal cells of the body [55]. It was also suggested that apoptosis incapable tumor cells (including these lacking TP53 and RB proteins) retain the ability to senescence while remaining sensitive to chemotherapeutic agents [60].

As it was mentioned, some drugs lead to the senescence of cancer cells by destroying DNA, mainly by causing single- and double-strand breaks [61]. This shows that senescence of tumor cells, stress-induced premature senescence (SIPS), and replicative senescence in physiological cells can be based on similar mechanisms. On the other hand, despite the accumulation of cytogenetic changes in the telomeres of breast cancer cells treated with doxorubicin, no telomere shortening was observed. This suggests that drug-induced cellular ageing occurs in a telomere-independent fashion [62].

Interestingly, there are contradictory data about the ability of drug-induced senescence and the expression of cell cycle inhibitory proteins. Recently, it was suggested that intact TP3 function is necessary for topoisomerase I-induced G2-M arrest [63]. On the other hand, senescence was induced by a variety of chemotherapeutic agents in the TP53-null, p16-deficient human non-small cell H1299 carcinoma cells [64]. Another study showed that as many as 20% of tumors that retain the ability to become senescent in response to chemotherapy showed mutated TP53 [65], while still others indicated that TP53-dependent senescence was induced [66], as exemplified by the induction of senescence in TP53 wild-type MCF-7 breast cancer cells exposed to doxorubicin [62]. In colon cancer cells treated with 6-anilino-5,8-quinoline quinone, the induction of p21^Cip1^, which can serve as an ageing promoter independent of TP53 signals, was observed [67]. However, in kidney cancer cells treated with sunitinib, the mechanism of cellular senescence was similar to that involving TP53, but without p21^Cip1^ contribution [68]. To study the role of p16^INK4a^ in cell senescence, in turn, osteosarcoma cells were designed that lacked this inhibitor, which resulted in impaired cell ageing [69]. An important role of p16^INK4a^ has also been observed in cisplatin-resistant NSCLC. In these cells, increased sensitivity to low doses of cisplatin was noted, which was accompanied by an increase in cell senescence after transfection with a construct encoding the full p16^INK4a^ sequence [70].

## 4. Senescence Induction as a Side Effect of Treatment with Regular Chemotherapeutics

As already mentioned, one of the most popular drugs that induce senescence is doxorubicin, which is believed to be a promising and effective therapeutic agent among oncological patients, mostly breast cancer patients [71, 72]. Studies on cell cultures and animal models showed that doxorubicin being a DNA intercalator is an apoptosis- and senescence-inducing agent, impairs cell division, and induces oxidative stress in cancer cells [71, 73]. It is proved that doxorubicin has also an adverse impact on noncancerous cells in the human body. For example, cardiomyopathies are usually observed in patients treated with doxorubicin. It is caused by the fact that doxorubicin induces senescence not only in cancer cells, but also has an unfavorable effect on cardiac progenitor cells (CPCs) [74]. Mouse mesenchymal stem cells are also observed to become senescent under treatment with doxorubicin [75]. Such observation may indicate that the implementation of doxorubicin in cancer patient treatment can be followed by inducing senescence not only in cancer cells but also in physiologically healthy cells, which are highly important in regenerative processes in the human body. Such impairment may be unfavorable for patients after chemotherapy because the recovery process is not efficient [74, 75]. Cancer cells are able to modulate the immune system and therefore inhibit the activation of effector immune cells through producing immunosuppressant factors. It lets cancer cells remain unidentified by the immune system, and the antitumor defense is ineffective [76]. Considering the fact that senescent cells gain the specific secretory phenotype-promoting inflammation, they are more likely to be targeted by immune system cells. Secreted cytokines play a role in the recruitment of immune system cells and lead to their activation [77]. Despite the possible adverse effects of doxorubicin in cancer patients, it still gives the advantage to fight cancer [78]. Cells that become senescent after treatment with doxorubicin are believed to be more susceptible to immune cell-mediated cytotoxicity. Inao et al. conducted the experiment in which they observed that after treatment with doxorubicin, breast cancer cell lines such as MDM-MB-231 and BT-549 had increased sensitivity to CD8+ and CD4+ T cells. Additionally, MDM-MB-231 cell line treated with doxorubicin was more sensitive towards antibody-dependent cellular cytotoxicity (ADCC) by natural killer (NK) cells when compared with untreated cells [73]. Therefore, inducing senescence in cancer cells may lead to making them targetable for effector immune cells and afterwards eliminating tumor cells from the body. A similar strategy is used in therapies with senolytic drugs, which induce apoptosis in senescent cells [79]. Unfortunately, doxorubicin is not specific only to cancer cells, and hence, it leads to increased susceptibility to immune response pathways in all cells that lost their proliferative potential. It results in numerous multi-organ side effects in patients [80]. However, such an approach is believed to be promising because senolytic combinations seem to be safer and more effective [78, 81]. Further studies have to be conducted to establish the best and the most effective doxorubicin combination in anticancer treatment.

## 5. To Get Rid of Senescent Cells

**Figure 1:**
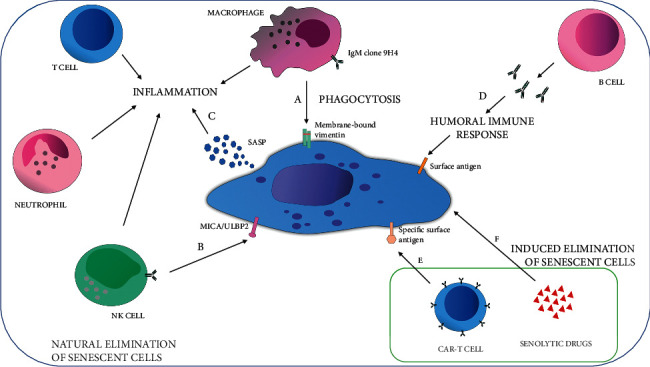
Processes of elimination of senescent cancer cells from the human body. Blue rectangle: natural elimination of senescent cells. (a) Recognition of membrane-bound vimentin on senescent (normal and/or cancer) cells' surface by macrophages *via* IgM clone 9H4 and phagocytosis of senescent cells. (b) Recognition of MICA or ULBP2 ligand on the surface of senescent cancer cells and elimination of them by NK cells. (c) Recruitment of NK cells, macrophages, neutrophils, and T cells *via* SASP factors and removal of both normal and neoplastic senescent cells by the immune cells. (d) Production of antibodies associated with humoral immune response by B cells and binding of antigens on the surface of senescent normal/cancer cells; green rectangle: induced elimination of senescent cells. (e) Elimination of senescent cancer cells by senolytic drugs such as ABT-263. (f) Recognition of specific surface antigen by CAR-T and removal of senescent cancer cells by means of CAR-T immunotherapy.

Senescence often plays an important role in many physiological and pathophysiological processes in human organisms. Induction of senescence may occur during embryogenesis, restoration of tissue integrity, or wound healing, and it is a barrier to the neoplastic transformation of cells [82]. However, the accumulation of senescent cells could affect the proper functioning of the human body and result in, e.g., organ dysfunction. Moreover, elements of senescence-associated secretory phenotype such as inflammatory cytokines may lead to the formation of chronic inflammation and consequently to the development of age-related diseases. To avoid adverse effects of senescence induction, the accumulated senescent cells are eliminated within the organism by the immune cells through various mechanisms (Figure 1) [82, 83]. So far, it is not immediately obvious what happens to senescent cells *in vivo*. In the available literature, there are reports suggesting that the immune system is responsible for the removal of both normal and cancer senescent cells. Macrophages are likely to play a key role in this process. It is speculated that senescent cells, just as apoptotic ones, have specific receptors on the surface that are recognized by IgM antibodies, which ultimately leads to phagocytosis of these cells by macrophages [84]. Recent studies indicated the IgM clone 9H4 is able to bind an oxidized form of membrane-bound vimentin on the surface of senescent human fibroblasts. Probably, senescent cells are recognized by antibodies through the agency of the membrane-bound vimentin and then phagocytized by macrophages (Figure 1(a)) [84, 85]. The natural killer cells also may take part in eliminating senescent cells from the human body. They are supposed to bind specific ligands, including MICA or ULBP2, occurring on the surface of senescent cells, which leads to clearance of these cells (Figure 1(b)). It is worth mentioning that the indicated ligands are more often found in the membrane of neoplastic cells than in normal cells [86, 87]. In addition, during a study conducted on a mouse model of liver carcinoma, in which senescence was induced through the increase in expression of TP53 protein, it was proven that the activity of the TP53 suppressor may promote the secretion of NK cell-recruiting chemokines by tumor cells. The obtained results indicate that the expression of TP53 and factors of SASP such as chemokines enable the effective elimination of senescent cancer cells by NK cells [87]. The SASP elements secreted by senescent cells are able to recruit not only natural killer cells but also other immune cells, i.e., macrophages, neutrophils, and T lymphocytes (Figure 1(c)). The accumulation of the indicated immune cells within the environment of SASP factors contributes to inflammation development. It is suggested that the inflammatory process mediated by SASP enables the removal of senescent cells *via* the immune system and tissue regeneration [83]. Analyzing the mechanism of senescent cell removal, the role of B lymphocytes should not be omitted. The B cells may eliminate senescent cells through the production of antibodies participating in the humoral immune response (Figure 1(d)). However, it is not entirely clear in response to which cell surface antigen B lymphocytes release the antibodies [86].

Induction of senescence during anticancer therapy acts as a two-edged sword. Irreversible inhibition of the cancer cell proliferation prevents tumor development and increases the effectiveness of oncological treatment. On the other hand, senescence may contribute to the progression of neoplastic disease, e.g., *via* senescence-associated secretory phenotype and stimulation of proliferation of non-senescent subpopulation of cancer cells. The elimination of senescent cancer cells seems to be a reasonable solution to achieve the desired therapeutic effect. Therefore, it is extremely important to understand the natural mechanisms of removing senescent cells from the body under physiological conditions, as well as the clearance pathways of senescent cancer cells. This knowledge may allow for the development of effective methods of senescent tumor cell elimination and thereby increase the chances of successful anticancer therapy.

## 6. Drug Combinations: Senolytic Agents in a Second Phase of Synthetic Lethality Approach

Due to the dualistic role of senescence, the usage of senolytic drugs may lead to a disruptive discovery for increasing the effectiveness of TIS. The senolytics are pharmacological agents responsible for the selective elimination of senescent cells by targeting biochemical processes essential for the existence of these cells (Figure 1(e)). ABT-263, a specific inhibitor of the antiapoptotic proteins BCL2 and BCL-xL, is an example of a strong senolytic drug. It can remove senescent cells independently of cell types by apoptosis induction in senescent cells. It was also proven that the treatment of p16-3MR transgenic mice (earlier treated with body irradiation to promote senescence) with ABT-263 agent successfully impacted the removal of senescent bone marrow hematopoietic stem cells and senescent muscle stem cells [28, 30]. In addition, combinations of senolytic agents are also used in therapy (not only antineoplastic). The first proposed combination of senolytics was the combination of dasatinib and quercetin (D + Q). These drugs selectively remove senescent cells through blockage of the senescent cell antiapoptotic pathways (SCAPs). The SCAPs protect senescent cells from their own SASP elements, which can induce apoptosis in these cells [88, 89]. It was proven that *in vitro* the combination of D + Q could be senolytic for mouse embryonic fibroblasts [90]. Moreover, the results of the first clinical trial of dasatinib and quercetin verified that the combination may improve the physical function of individuals suffering from idiopathic pulmonary fibrosis (IPF), which is a severe and deadly disease associated with senescence. In the open-label phase 1 pilot study, it was also suggested that D + Q treatment of patients with diabetic kidney disease (DKD) resulted in a decrease in the adipose tissue or skin epidermal (expressing p16^INK4a^ and p21^CIP1^) senescent cells and elimination of senescence-associated secretory phenotype factors such as interleukin IL-1*α* or IL-6 [88].

Currently, the senolytic combinations could be used also in anticancer therapies. These combinations are proposed based on the strategies of the synthetic lethality approach [91]. Synthetic lethality is the concept assuming that the cooccurrence of mutations in two different genes leads to cell death. Such a concept also points out that when these mutations occur separately, the cell would remain its viability [92]. Synthetic lethality is mostly observed for loss-of-function alleles, but sometimes it can also relate to gain-of-function alleles. An example of synthetic lethality in the case of loss-of-function alleles is a situation when two different proteins are crucial elements in two parallel and inseparable pathways. In case of losing only one of these genes, the cell can be still viable, because the function of the lost allele would be somehow compensated by a non-mutated allele, but in case of losing both these alleles, the cell would not be able to maintain viability. On the other hand, gain-of-function alleles are less likely to be observed in synthetic lethality, but there are some cases when the overexpression of one gene is balanced by the other gene. In such a situation, the loss of this second one would be lethal for the cell [93]. In general, the synthetic lethality concept is based not only on the activation of oncogenes as targets but also on suppressor genes, genes that are associated with DNA repair mechanisms or/and regulation of cancer cell metabolism [92].

Relying on the synthetic lethality approach, it is also possible to induce senescence. Thus, not only apoptosis but also senescence may be involved in the synthetic lethality interactions *in vivo.* This was verified for the first time between genes encoding the DNA repair and recombination protein RAD54-like (Rad54) essential for homologous recombination (HR) and the poly(ADP-ribose) polymerase 1 (PARP1) responsible for DNA repair in Ptc1 heterozygous mice with developed medulloblastoma. The mutations leading to the inactivation of both alleles of PARP1 and Rad54 genes resulted in proliferation arrest and senescence induction in cancer cells through activation of the TP53-p21 pathway and inhibited tumor growth [94]. Nevertheless, not all experiments confirm the antitumorigenic role of p21. In accordance with that, p21 is not mutated in cancer cells and can participate in senescence reversion [95].

A novel approach in the treatment of cancers characterized by the presence of BRCA1/2 mutations, such as ovarian and breast cancer, was also analyzed. One of the most promising and effective drugs is olaparib, which is an inhibitor of PARP1. Another example of PARP inhibitors is talazoparib. Both drugs are trapping PARP-DNA complexes, which makes DNA repair impossible [92, 96]. PARPs are strongly associated with repair mechanisms of the DNA damage, while BRCA1/2 mutations are involved mostly in HR. Considering the fact that BRCA1/2 mutations lead to impairment of HR mechanism, cancer cells are more prone to induction of the synthetic lethality. Therefore, the occurrence of the BRCA1/2 mutation and parallel treatment with only olaparib mainly result in cell death but also senescence induction or higher susceptibility to the anticancer drug. On the other hand, the PARP inhibitors in combination with BCL2 inhibitors were observed to have a synergistic effect in the therapy of ovarian cancer. This combination is much more preferential to induce apoptosis in ovarian cancer cells than using PARP and BCL2 family inhibitors used in monotherapy [91, 92]. However, ovarian cancer cells in which senescence was induced by means of PARP inhibitor may escape cell cycle arrest. Events like that may be highly unfavorable. Cancer cells that were able to regain proliferative activity are suspected to have increased tumorigenic and metastatic potential [81, 91].

**Figure 2:**
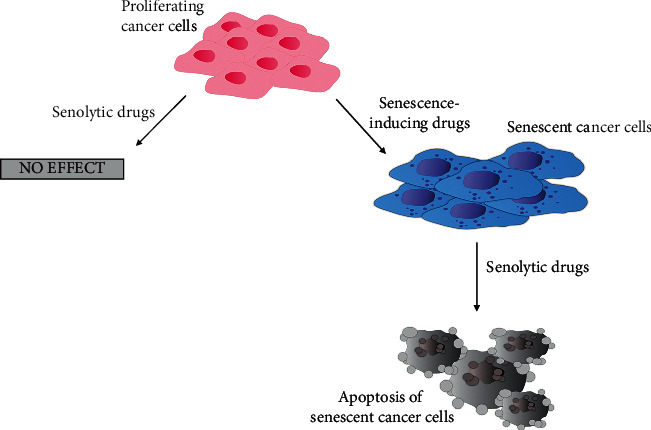
Combination of senescence-inducing drugs and senolytic drugs compared with single therapy with senolytic drugs. A drug combination in which a senolytic agent is used in a second phase of synthetic lethality approach seems to be the most beneficial approach for cancer patients.

To avoid the risk of reversibility of senescence or negative effect of SASP activity, it seems to be important to eliminate senescent cells from the body during the treatment. It may be possible employing the usage of senolytic agents such as ABT-263. The one-two punch strategy is believed to increase the effectiveness of cancer treatments based on TIS. It has been demonstrated that the synthetic lethality approach can be used, e.g., to treat breast and ovarian cancer patients with the agents responsible for senescence induction within the first step of the treatment, and, in a second phase, senolytic agents that target senescent cells leading to their apoptosis and therefore reducing the number of cancer cells (Figure 2). Induction of senescence of tumor cells on its own would not result in eliminating cancer cells from the patient's body, while the usage of only senolytic drugs against cancer cells, which do not exhibit features of senescence, would not be effective. In the study conducted by Fleury et al., it was verified that olaparib may induce senescence in ovarian and breast cancer cells and the inhibitor of antiapoptotic proteins BCL2 and BCL-xL (ABT-263) was able to eliminate senescent cancer cells *in vitro*. They also proposed another approach that was associated with promoting additional DNA damage in cells already treated with PARP inhibitors, which resulted in synthetic lethality in tested cells [91, 97].

Interestingly, in 2020 for the first time a senolytic therapeutic concept of eliminating senescent cells *via* CAR-T was developed (Figure 1(f)). The genetically modified T lymphocytes target senescent cells by recognizing the urokinase-type plasminogen activator receptor (uPAR)11 as a cell surface protein induced during senescence. Amor et al. showed that uPAR-specific CAR-T efficiently ablate senescent cells *in vitro* and extend the survival of mice with lung adenocarcinoma that are treated with a senescence-inducing combination of drugs [98].

Senolytic drugs and inducing synthetic lethality are promising methods in cancer therapies. They give hope of improving current treatments and their outcomes among oncological patients. Although these approaches have some disadvantages and restrictions, the combination of synthetic lethality and senolytic drugs seems to be even more effective and safer for the patients as it lets us target-specific cells like cells with a particular mutation. As mentioned before, currently there is only one combination being under *in vitro* and *in vivo* investigation on breast and ovarian cancer models, which gives favorable results. Olaparib and ABT-263 used in combination are the proof of concept that researchers should focus on inducing synthetic senescence in cancer cells with the following usage of senolytic drugs.

## 7. Oncogene-Induced Senescence

Even though research on senescence has been conducted for over 50 years, its complicated mechanism is still not fully understood, in neither normal nor tumor cells. Moreover, depending on the factor initiating senescence, several types of senescence have been identified [99]. Surprisingly, according to studies from the last decade, oncogene activation may be responsible for an irreversible cell cycle exit in primary cells, called oncogene-induced senescence (OIS). The mechanism of OIS usually requires activation of DNA damage signals induced by replication stress [100]. Unlike drugs that directly damage DNA, the action of oncogenes must be multistage to contribute to premature senescence. Early data suggested that quick proliferation may simply lead to telomere erosion and OIS is just gaining faster Hayflick limit, and indeed, in normal cells, senescence is usually associated with shortening of telomeres [101]. However, cancer cells have demonstrated the presence of telomerase, which prevents the shortening of telomeres, precluding this type of senescence undermining this hypothesis at least for cells with high telomerase activity [102]. The switch between pro-proliferative *versus* pro-senescent actions of oncogenes is not defined. In general, the growth of the cells formally means their enlargement. It means that oncogenes are still activating many processes requiring building new structures after triggering senescence, but these processes are dedicated to building one large cell, not a colony of smaller ones. It is very important to realize that even within selected tumor one subpopulation of cancer cells becomes senescent, whereas other proliferates quickly in response to the same oncogene activations but apparently proliferation prevails (at least *in vivo)* [103].

Oncogene-induced senescence may be induced by the activation of oncogenic RAS and BRAF proteins or the loss of the suppressor protein PTEN or NF1 [104]. Among the first most important observations was the fact that RAS^G12V^ induces senescence in fibroblastic cells. The RAS pathway can activate p14^ARF^ expression, which makes TP53 insensitive to MDM2 regulation and finally stops cell division. Inhibition of TP53 in these cells changes the consequences of RAS^G12V^ action. Therefore, the question should be asked whether the restoration of TP53 activity in cells with the RAS mutation will make this oncogene an inhibitor of the neoplastic process. If this is true, blocking the RAS pathways at all costs would be not as effective as reactivating its pro-senescent role by restoring TP53 operation. The induction of senescence in tumor cells depends largely on two connected tumor suppressor pathways: TP53 and RB/p16^INK4a^. Activation of TP53 and RB may take place through phosphorylation, protein stabilization, and protein-protein interactions [105]. In the case of senescence or cell cycle arrest in general, TP53 increases the concentration of p21 protein, which in turn inhibits the cyclin/CDK complexes [106]. After stopping cell division, the expression of TP53 and p21 decreases, while p16^INK4a^ (another cyclin-dependent kinase inhibitor) increases, suggesting that p16^INK4a^ protein is responsible for the permanent arrest of cell proliferation [107].

Another important protein in the senescence process is RB. The activity of this protein is regulated by cyclin-dependent kinases (CDK2, CDK4, and CDK6) [108]. The RB protein is a repressor of genes involved in DNA replication (RB inhibits E2F transcription factors, which are essential for cell proliferation) [105]. TP53 and RB are absent in many cancer cells, but still these cells become easily senescent *in vitro* but it is not obvious whether this is OIS. Intriguingly, the loss of TP53 might not only bias oncogene action from senescence to proliferation but also form apoptosis to proliferation. Indeed, the hyperactivation of CDK and lack of RB can cause apoptosis TP53- and p14^ARF^-dependent. According to subsequent studies, senescence caused by the loss of the suppressor protein PTEN is another type of senescence and it is worth distinguishing this type among OIS [109].

Activated oncogenes force the cell to replicate in an uncontrolled manner, which leads to DNA replication stress [110]. There is no doubt that the signaling context determines whether RAS and probably many other oncogenes cause senescence or proliferation. *In vitro*, it is very difficult to stabilize the proliferation of neoplastic cells. At present, it is difficult to establish why *in vitro* the pro-senescent arm of oncogene actions begins to dominate rather than the pro-proliferative one, but apparently, it is so.

## 8. Senescence as a Culprit of Failure in Stabilization of Cancer Cell Cultures

It was mentioned that senescence of tumor cells appears as a double-edged mechanism with both tumor-supported and tumor-suppressive roles. It is therefore not surprising that many researchers analyzed the role of this intriguing phenomenon under *in vitro* conditions. In 2014, Stoczynska-Fidelus et al. conducted analyses of glioblastoma cells taken from 57 patients in various types of *in vitro* conditions and turned out that these cells could be propagated only for a limited number of passages [111]. Additionally, it was turned out that normal cells surrounding neoplastic cells and infiltrating the tumor (further studies indicated that these cells were glioma-associated stromal cells, GASCs) may proliferate *in vitro* longer than cancer cells and overgrown the latter ones. Numerous ongoing studies on negative *in vitro* selection of tumor cells have been carried out since 2011 when Witusik-Perkowska et al. showed that 3D cultures enable to maintain glioblastoma cells in proliferating state longer than in monolayer [112]. Piaskowski et al. demonstrated that glioma cells with a mutation in the *IDH1* gene cannot be maintained under standard culture conditions [113]. Elucidation of the phenomena responsible for failure in the stabilization of some tumor cells constituted an important issue for many researchers who have been focusing on the difficulties with glioblastoma (GB) cultures [114]. The loss of epidermal growth factor receptor (*EGFR)* amplification already in first passages was observed in all analyzed *in vitro* conditions, even in 3D neurospheres or in cell xenografts [115]. This phenomenon entails considerable consequences; e.g., genetically modified cell lines that often do not reflect the complexity of the extrachromosomal nature of amplicons are often applied for research on compounds with antineoplastic potential [116].

We suggested that cellular senescence may constitute the major culprit of failure in the stabilization of GB cultures, especially with amplification of oncogenes such as *EGFR*, epidermal growth factor receptor variant III (*EGFRvIII),* and platelet-derived growth factor receptor (*PDGFR)* [111]. At that time, it seemed unlikely that tumor cells are more prone to senescence than normal cells in any condition. Combining several techniques (double immunocytochemical staining, BrdU incorporation assay, senescence-associated *β*-galactosidase assay, and real-time *in vitro* observations), it was possible to distinguish normal cells infiltrating the tumor from cancer cells and contribute to the hypothesis that senescence is indeed the major culprit for the failure in the stabilization of tumor cells *in vitro*. Moreover, it was demonstrated that *TP53* mutation and heterozygous *CDKN2A* deletion, considered as mutually exclusive, cooccurred in more than half of stabilized lines [111]. Even more interesting was the fact that although *EGFR*, *EGFRvIII,* and *PDGFRA* amplification was detected in surgical specimens, it was not preserved in cell cultures. These observations in stabilized cultures may be caused by the wide range of phenomena; e.g., senescence may be related to mitotic catastrophe due to the fact that the latter was simultaneously detected under the same conditions [111]. It has to be emphasized that at least two hypotheses should be considered to explain such phenomena. Firstly, massive senescence resulting from mitotic catastrophe is a process responsible for the inability to maintain cells with the amplification of oncogenes under *in vitro* conditions. Secondly, these cells stabilize *in vitro* but with previous loss of amplicons acting as an adaptive mechanism. Indeed, in 2019 Janik et al. showed that SA-*β*-Gal-positive cells were in high percentage polynucleated and undergo abnormal mitoses in early passages [23]. Multipolar spindles, as well as polynuclear cells with asymmetric distribution of phosphorylated histone 3, were easily detected in early passages of glioblastoma tumor cells. The coexistence of mentioned features with tumor cell markers showed beyond a reasonable doubt a link between abnormalities in mitosis of tumor cells with senescence of these cells *in vitro*.

Identifying such idiopathic senescence opened the way to research the details of the mechanism responsible for this phenomenon. Analyses were subsequently continued on a group of glioblastomas expressing IDH1^R132H^ protein. Introduction to the market of a unique specific antibody directed against isocitrate dehydrogenase 1 (IDH1) mutated in codon 132 (IDH1^R132H^), a specific marker of glioblastoma arising from lower-grade tumors [117, 118] and application of this antibody in described method are enabled to prove that senescence occurs in cancer cells, not normal cells infiltrating the tumor [119]. Furthermore, the applied methodological approach enabled to reveal that stem-like cells may undergo *in vitro* senescence, as confirmed with double IDH1^R132H^ and SOX2 staining [120].

Further, it was confirmed that the abovementioned phenomena are not specific only to primary glioblastoma cultures but rather universal. Firstly, molecular analysis of cancer cell line databases demonstrated the universal issue of lack of appropriate experimental models; e.g., there is only one cell line with endogenous expression of IDH1^R132H^-HT1080 [121–123]. This cell line was derived from fibrosarcoma, but mutations in the *IDH1* gene are characteristic for several other cancer types, such as central nervous system (CNS) tumors or acute lymphoblastic leukemia (the most common one) [124]. Secondly, senescence was easily detected in a number of experimental models—primary cultures of prostate, breast, and colon cancer, as well as stable cell lines of various cancer types [103]. Analysis of stable cell lines also provided some intriguing results. Populations of SA-*β*-Gal-positive cells were detected in NCI-H460, SK-MEL28, NCI-H1975, and MCF-7 cell lines, while no traits of senescence were observed in MDA-MB-468 and SW962 lines [103]. Differences between cell lines require further studies, particularly in view of the fact that senescence may play a pro- or antineoplastic role. It is hard, here, not to mention the aspect of immortality that also seems to be very interesting in the context of cellular senescence. It needs to be considered whether tumor, understood as a set of clones, has the potential to become immortal, while single neoplastic cells within the tumor may have various functions, which exhibit traits of senescence and/or be mortal.

What is equally important, as it was mentioned, SA-*β*-Gal activity and formation of SAHF are two main senescence markers. However, among all analyzed cell lines and primary cultures, any single cell positive for both SAHFs and SA-*β*-Gal activity was not detected. On the other hand, SAHF presence may be characteristic for only one out of various mechanisms inducing the senescence phenomenon. It is evident that the formation of these structures is most often associated with OIS (*via* activation of RB/p16^INK4a^ pathway) [125].

## 9. Mitotic Catastrophe and the Choice between Programmed Cell Death and Senescence

**Figure 3:**
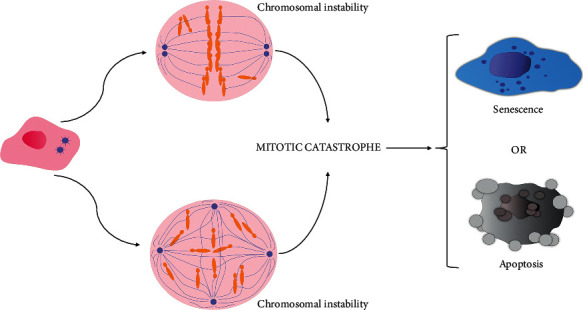
Exemplary cases of a mitotic catastrophe associated with abnormal chromosome segregation and/or chromosomal instability. A mitotic catastrophe can result in cell death or senescence.

Another intriguing phenomenon linked to senescence is a mitotic catastrophe. Eukaryotic cells developed a mechanism leading to the elimination or senescence of cells showing DNA damage during mitosis or are suspected of ending mitosis with chromosomal aberrations [126, 127]. Indeed, very often this phenomenon is a type of cell death. However, mitotic catastrophe may also end in senescence (Figure 3), which means that when considering it among cell deaths, it should be clarified what exactly is meant [128]. If cancer development is considered, senescence seems to be a type of compromise. If loss of cell division ability is indeed irreversible, such cell cannot become a tumor seed. However, this cell with an altered genome may still be useful in the normal tissues by, for example, secreting important proteins, but, unfortunately, among proliferating tumor cells they will play the role of tumor support. Megakaryocytes represent a very interesting example here. Megakaryocytes produce normal platelets despite chromosomal changes that would be considered serious aberrations in other cells. Moreover, as it was mentioned, senescent cells have the ability to produce many substances, mainly secretory proteins, for example, the extracellular matrix proteins [129].

It is generally recognized that mitotic catastrophe is triggered by abnormal mitosis. Under normal circumstances, the spindle assembly checkpoint (SAC) plays a role in the process ensuring a smooth transition from metaphase to anaphase. The way chromosomes are attached to the kinetochores is monitored, and until anchoring is properly secure, the anaphase will not begin. If this process is correct, the CDC2-CDK1 gene product activates the E3 ubiquitin ligase and APC (anaphase-promoting complex) leading to ubiquitination and degradation of cyclin B, which is responsible for keeping CDK1 active. After CDK1 inactivation, securin is degraded, which releases a separase that prevents the separation of sister chromatids. Finally, degradation of separase leads to anaphase [130]. Following APC-mediated degradation of cyclin B and inactivation of CDK1, a so-called mitotic exit can occur. It can sometimes occur without cytokinesis, or even anaphase, which is referred to as mitotic slippage [131].

The impact of DNA damage detected at the earliest stages of mitosis (before turning off transcription) has an in-depth studied effect. DNA damage activates a cascade of events of DNA-PK/ATM/ATR kinases, leading to the inactivation of the cyclin B1/CDK1 complex, which, as described later, is crucial for the activation of apoptosis. CHKs phosphorylate and inactivate CDC25C, thus preventing the activation of CDK1 (*CDC2* gene). CDC25 is a CDK1-activating phosphatase [132]. The high levels of 14-3-3 sigma, the growth arrest and DNA damage-inducible 45a (GADD45a), and p21 are triggers of that process. 14-3-3 sigma sequesters CDC25C into the cytoplasm, thus preventing the activation of the cyclin B1/CDK1 complex in the nucleus; GADD45 binds to and dissociates the cyclin B1/CDK1 complex (*CDC2* gene), and p21 inhibits CDK1 kinase activity [133]. After the prolongation of mitosis blockade, apoptotic proteins are then activated, and CDK1 (a *CDC2* product) blocks antiapoptotic proteins. Thus, after an extended time, CDK1 successfully activates a mitotic catastrophe mainly related to apoptosis, or in the absence of these proteins, mitotic slippage occurs [134]. In the case of mitosis, time acts as a factor reducing the concentration of cyclin B and this leads to the choice of senescence over apoptosis [135].

In the case of chromosomal aneuploidies, it is difficult to imagine an analogous repair system as in the case of DNA damage. There is no typical repair system for chromosome loss as nucleotide deletion in DNA [136]. Typical reversible DNA damage can only be compared to the rearrangements of chromosomes when their separation begins. In general, the sequence of events leading to programmed cell death or senescence during mitosis may be similar to events observed after detecting unrepairable or for too long unrepaired DNA damage during the S phase or replication. However, due to the specificity of mitosis, other protein characteristic of chromosomal interactions, apart from, CDK1 and cyclin B (they constitute a common denominator), participate in it. The whole process of controlling proper mitotic apparatus assembly is very complex, and this complexity is important because mitotic catastrophe is very often determined not so much by the detection of an aberration during anaphase, but by the fact that the process of correcting the chromatid alignment takes too long [130].

In the introduction to this paragraph, we described mitosis under normal conditions in the context of cyclin B degradation and CDK1 inactivation. Alternatively, if the spindle assembly checkpoint detects abnormalities in the mitotic spindle, it delays chromatid separation until the sister kinetochores are stably attached to the microtubules of the mitotic spindle. This process gives the cell time to correct the wrong kinetochore-microtubule connections and to avoid possible disturbances in the chromosomal composition that can lead to carcinogenesis. The whole process does not exactly repair but is rather a correction of the errors in the machinery preparing transport of chromatids to future sister cells [137]. A protein playing a very important role at this stage is Aurora B. It is the catalytic subunit of the passenger chromosomal complex, which also contains the inner centromere protein INCENP scaffold protein and survivin and borealin [138–140]. Aurora B is located in centromeres and kinetochores in prometaphase. It ensures the ordering and subsequent segregation of chromosomes by promoting the disconnection of kinetochores poorly attached to the microtubules [141]. In eukaryotic cells, Aurora B is necessary to arrest mitosis in the presence of improperly attached kinetochores-microtubules or the presence of many unrelated kinetochores [142]. Participation of CHK1 or Aurora B in the correction of the so-called merotelic attachments is another important element of mitosis control [143].

As can be seen, the term “repair” in the context of cell division has a specific meaning. It means correction in the assembly of the elements involved separation of chromosomes [142, 144]. If abnormal chromosome positions are detected during mitosis and are not corrected, it is possible that after a temporary standstill, cells escape from this block through mitotic slippage. Mitosis is intermittent. There is a return to interphase, but as a consequence, a doubled number of chromosomes appear. Even several replication cycles with the disruption of mitosis leading to polyploidization can be observed [129, 145]. Sometimes, some of the cells that survive this stage start to perform the reverse process, diploidization, and can even return to mitosis terminated by cytokinesis [146]. After such events, other phenomena can occur again, e.g., programmed cell death during the G1 of the next cell cycle, or a mitotic catastrophe during the next mitosis, especially during the start of anaphase [130, 145]. The occurrence of apoptosis during G1, which begins with mitosis, shows that the study of the phenomenon of mitotic catastrophe may extend beyond the time of mitosis. The process by which programmed death occurs in the later stages of mitosis is sometimes described as an anaphase catastrophe [147].

Finally, mitotic slippage leads to more chromosomal alterations. In consequence of polyploidization, the elimination process of cells with genetic alterations from the pool of proliferating cells may be intensified. However, it is also believed that mitotic slippage is the most common way to escape the programmed death of cancer cells that have been subjected to chemotherapy that damages DNA or disrupts the function of the karyokinetic spindle. The potential use of the phenomenon of a mitotic catastrophe in therapy depends on the skillful influence on the course of the cell's fate during cell division [145, 148]. This is particularly important to realize that the consequences of senescence can be somehow beneficial to the cancer process, as can mitotic slippage, but not the consequences of programmed or any cell death [130]. It is proposed that the choice of programmed death depends on the level of cyclin B and proapoptotic proteins. If the concentration of cyclin B remains high for a long time, a relatively low concentration of proapoptotic proteins is enough for apoptosis to occur. However, if during prolonged mitosis, the level of cyclin B is decreased before the concentration of apoptotic proteins increases to ensure effective apoptosis, then higher and higher concentrations of apoptotic proteins are needed to achieve programmed cell death. So if the concentration of cyclin B decreases within the time determined by the cell clock, it may lead to mitotic exit or rather a mitotic slippage, or senescence instead of apoptosis directly related to mitotic catastrophe. This is because cyclin B is needed for the function of CDK1, which phosphorylates BCL2, BCL-x1, among others, thus facilitating programmed death [130, 149].

It should be recalled here that the mitotic exit opens the option of either senescence or the previously described mitotic slippage with polyploidization, which can lead to apoptosis in G1, or even to mitosis after depolyploidization. The choice between mitotic slippage senescence and apoptosis is coordinated during mitosis through the concentration of cyclin B apoptotic proteins and the duration of mitosis [150]. Some authors suggest that TP53 can play the role of a very important switch between programmed death and senescence during late mitosis. However, there are no clear explanations for such an assumption. Probably, it comes from the general TP53 role. TP53 gives time to restore a cell to normal status, whether it is DNA damage or some other type of stress, and if that restoration fails, apoptosis is achieved. However, the role of TP53 in the regulation of the mitotic catastrophe cannot be easily explained based on TP53 transcription factor activity because, during mitosis, the majority of the transcription and translation processes are blocked due to chromatin changes, but many operations of TP53 are quite dependent on them [151]. It is sometimes suggested that the active TP53 protein temporarily protects against the occurrence of the mitotic catastrophe because it can stop the cell in the initial stages of mitosis after which cytogenetic disturbances would occur and enable correction in an arrangement of chromosomes [152].

A mitotic catastrophe is the result of the inability to arrange chromosomes for too long. Alternatively, mitotic catastrophe can occur during anaphase, after the detection of very large malfunction or disorder in the mitotic spindle [153]. Lack of transcription during mitosis does not interfere with transcription-independent actions of TP53 and with the so-called post-slippage induction of apoptosis (G2 of the next mitotic cycle). During this time, the activity of Aurora A and Aurora B kinase increases. This prevents the continuation of the cell cycle by increasing the concentration of the p21 CDK blocker [154]. Naturally, earlier (pre-mitosis) elevation of B-cell lymphoma protein 2 (BCL2) concentration in the absence of TP53 may prevent programmed cell death, counteracting the effects of cyclin B1, or rather CDK1. TP53 always prepares the cell for subsequent events by controlling both BCL2 and B-cell lymphoma protein 2-associated X (BAX) levels and changing their ratio in favor of BAX. As a result, the consequences of the lack of TP53 protein are difficult to predict. Elimination or inactivation of TP53 protein may result in the inability to arrest mitosis longer and correct abnormalities in chromosome assembly. From this point of view, the lack of TP53 may contribute to a decrease in the number of mitotic catastrophes. However, chromosomal aberrations cumulated during unstopped by TP53 mitoses may increase the probability of mitotic catastrophe during the next mitoses. It is known that neoplastic cells are more sensitive to the phenomena causing mitotic catastrophe than many normal cells. For example, cancer cells are more likely to undergo mitotic catastrophe under the influence of taxanes than do fibroblasts [155].

It has been suggested that abnormal chromosome segregation may influence whether aneuploidy promotes or suppresses tumorigenesis. Low levels of aneuploidy can promote while high levels prevent neoplastic growth [156]. Interestingly, the deletion of the *CDKN1A* gene encoding p21 (expression of p21 is enhanced by TP53) correlates with mitotic catastrophe and is more often accompanied by programmed cell death than senescence [150]. This scenario seems to be a logical consequence of above-described mechanism.

One of the causes of OIS is DNA damage that results from excessive stimulation of proliferation. It is worth asking at this point whether the activation of oncogenes may facilitate a mitotic catastrophe and it seems likely. Faster proliferation rate correlates with more frequent DNA damage, including errors leading to loss of whole chromosomes, translocation, etc. The exact mechanism of this type of OIS is not well explained. Other than DNA, damage mechanisms are also considered. As already mentioned, RAS^G12V^ can induce apoptosis as long as TP53 is active in the cell because RAS induces p14^ARF^ expression. On the other hand, some publications suggest that during the mitotic catastrophe resulting from telomere erosion, TP53 is not needed. Discovering how it is possible could guide to turning on a mitotic catastrophe in TP53-independent manner during the therapeutic process. Cancer cells quite often have active telomerase. Either way, we can see that oncogene action can lead to chromosomal aberrations [157].

Chromosomal abnormalities are not always beneficial for the cancer cell. Most attention is paid to the fact that the loss of chromosomes or their fragments means the loss of tumor suppressors. However, the loss of fragments of chromosome can be the loss of the oncogenes inhibiting the neoplastic process. Mitotic catastrophe or typical apoptosis after massive irreversible DNA damage can be a necessity even in the case of cancer cells. Cancer cells may therefore be more predisposed to a mitotic catastrophe than normal cells, and this is the result of a change in their biology, e.g., accelerated division rate. Additionally, neoplastic cells very often show disturbances in the number and structure of centrosomes involved in the regulation of chromosome segregation, which may contribute to the appearance of an increased number of chromosomal aberrations [158]. Treatments such as olaparib-dependent show that even cancer cells after crossing certain thresholds of DNA damage become apoptotic.

So far, very little attention has been paid to the role of extrachromosomal amplicons during studies of a mitotic catastrophe or DNA damage in general. There are many questions here. Whether amplicons are susceptible to all DNA damage repair systems? This seems very likely since they have chromatin. However, the next question is more difficult. Can asymmetric distribution of amplicons during mitosis generate an effect similar to the uneven distribution of chromosomes—arrest mitosis during an attempt to arrange them symmetrically? It seems very unlikely since amplicons cannot attach kinetochores and are likely to be separated quite randomly after tethering to chromosomes (described in detail later). Nevertheless, DM amplicons retain some ability to remain in the cell nucleus after mitosis. Amplicons contain chromatin, e.g., S/MARs (scaffold/matrix attachment regions). These elements are the points of DNA attachment to chromatin proteins and serve to organize its structural domains. For this reason, it has been concluded that double minute (DM) amplicons containing multiple S/MARs attach to chromosomal structures allowing tethering. The uneven distribution of amplicons does not seem so far to be a signal to suppress mitosis into anaphase. However, it is not certain whether there is any possibility of detecting their uneven distribution in mitotic cells at all. It is plausible to hypothesize that their drastically uneven distribution will trigger apoptosis, e.g., during anaphase or cytokinesis. If there is a system sensing to this type of DNA localization, then because extrachromosomal amplicons are present in most types of cancer cells, triggering dependent on amplicon mitotic catastrophe could be a breakthrough in cancer therapy. Experiments on viral DNA, which readily integrated with DM amplicons, gave very interesting results. Therefore, amplicons seem to resemble episomes, which are more and more willingly used in biotechnology as non-integrating DNA elements, which can induce, e.g., reprogramming of mature cells into induced pluripotent stem cells (iPSc). The mechanisms of episome transport during mitosis and their attachment to chromatin are not well understood. Results suggest that amplicon DNA (S/MAR sequences) could interact with chromosomal proteins, mainly histones, and chromosomal proteins. Although there is no indication that such interactions could delay the mitotic process, and thus contribute to a mitotic catastrophe, still it is undoubtedly also a neglected direction of mitotic catastrophe research.

## 10. The Theory of Senescence Reversibility

It is worth mentioning that SAHF presence is correlated with the irreversibility of senescence, among other things since the formation of these structures hinders access of transcription factors (such as E2F) to genes involved in the cell cycle. The lack of SAHF in SA-*β*-Gal-positive neoplastic cells may suggest that the definition of senescence concerning these cells requires redefinition. So far, Serrano provides evidence for senescence reversibility by demonstrating the potential of senescent cells to reenter the cell cycle [159]. Skin cancer cells exposed to ionizing radiation were proven to become senescent, presenting markers typical for this phenomenon: SA-*β*-galactosidase activity and heterochromatin foci. Observed cells once again became invasive and metastasize [160]. Regarding replicative senescence, and a well-known statement that telomerase does not reverse the senescence growth arrest, Beauséjour et al. showed that, depending on the expression of p16^INK4a^, senescence is not necessarily irreversible and cells with high levels of p16^INK4a^ at senescence failed to proliferate upon TP53 inactivation or RAS expression, although they reentered the cell cycle without growth after RB inactivation [12, 161]. These results indicate that the senescence response to telomere dysfunction may be reversible and is maintained primarily by TP53. However, p16^INK4a^ provides a dominant second barrier to the unlimited growth of human cells. Finally, phenomena such as reprogramming of fully mature somatic cells to pluripotent stem cells have been reported.

In general, as already mentioned, it is realized that each type of senescence requires the activation of the suppressor proteins TP53 and RB, which regulate pathways responsible for cell division [162–165]. On the other hand, mutations in these suppressors occur in most, if not all, human cancers. Human cells that lose TP53 and RB functions are generally refractory to multiple senescence-inducing stimuli [166, 167]. These and other studies suggest that the senescence response suppresses the development of cancer in mammals [162, 168, 169]. Presumably, TP53 recognizes dysfunctional telomeres as damaged DNA and acts on the p21 CDKI to increase its expression, which prevents RB phosphorylation and inactivation, inducing a senescence response [170]. However, in many human cells, the possibility of proliferation was observed despite short telomeres, due to the inactivation of TP53 or RB (e.g., by viral oncoproteins or antisense oligonucleotides), thus extending replicative lifespan [171, 172].

Even though the TP53 and RB pathways interact with each other, they can also induce cellular senescence by acting separately. This is confirmed by the fact that senescent cells show increased activity of another CDKI—p16, which is also involved in the control of RB activity [107, 173, 174]. Some human epithelial cells senesce with relatively long telomeres and high expression of p16^INK4a^, showing that p16^INK4a^ can inhibit cell proliferation through a mechanism other than TP53 [175, 176]. Besides, the ectopic expression of telomerase in these cells did not prevent the cells from going into replicative senescence, indicating that p16^INK4a^ expression and function are independent of telomerase status [175, 177]. Studies on human fibroblasts and epithelial cells show that depending on the expression of the RB regulator—p16^INK4a^, replicative senescence may be reversible and increased expression of telomerase does not halt senescence. Senescent cells with low p16 levels resumed strong growth after TP53 inactivation and limited growth after expression of the oncogenic RAS protein, while senescent cells with high p16 level did not resume proliferation after TP53 or RAS inactivation, but reentered the cell cycle without growth after RB inactivation. This proves that cellular senescence due to telomere dysfunction is reversible and largely dependent on TP53, but p16 is the second major barrier to unrestricted growth of human cells [161].

The inactivation of TP53 and/or p16^INK4a^/RB signaling pathways has occurred among the majority of clinical tumor specimens. Based on the aforementioned studies, cell leakage from TIS should be a common occurrence. In addition, two of the most studied cancer cell lines (TP53 wild-type), MCF-7 breast cells and HCT-116 colon cells, lack functional p16^INK4a^ [178, 179], which also suggests the frequent occurrence of the phenomenon of reversible senescence in cancer cells. However, in fact, in MCF-7 cells treated with a clinically relevant dose of adriamycin, which causes a widespread TP53-dependent senescence arrest in MCF-7 breast cancer cells, which is characterized by length-independent telomere dysfunction [62], a clonal-based proliferative recovery of mass cultures was observed [180]. A study carried out by Chang et al. on HCT-116 cells and HT1080 fibrosarcoma cells treated with doxorubicin points indicates a contrary position. They showed that HT1080 fibrosarcoma cells were able to divide after removal of doxorubicin from the environment; however, this was limited to only one or two cycles of the division [181].

**Figure 4:**
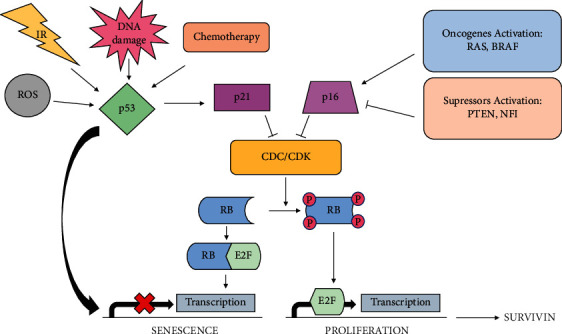
Regulation of proliferation/senescence status by TP53 and p16^INK4a^ activity in therapy-induced senescence (TIS), oncogene-induced senescence (OIS), and the reversibility of senescence. Senescence of cells can be induced by DNA-damaging factors such as IR, ROS, or chemotherapy, which inhibits RB phosphorylation and reduces the transcription of, *i.e.*, survivin, leading to senescence. Activation of oncogenes and inhibition of suppressors can control p16^INK4a^ activity, which consequently suppresses RB phosphorylation and also leads to senescence. Reduction in TP53 and p16^INK4a^ activity causes an increase in the CDC/CDK level, and finally, survivin level may lead to the exit of cells from arrest and restore the ability to proliferate.

Scientists from Daniel Wu's laboratory indicated repeatedly that the senescence induced by chemotherapy is reversible based on the H1299 model of non-small cell lung cancer with TP53 null/p16 deficiency, pointing to the role of CDC2 and survivin as a factor enabling escape from TIS (Figure 4) [182–184]. As one of the first shreds of evidence, H1299 cells were shown to be able to recover from senescence induced by the topoisomerase I inhibitor, camptothecin, which was associated with the overexpression of cyclin-dependent CDC2/CDK1 kinase [182]. It has also been indicated that the escape from senescence can be disrupted by CDC2/CDK1 kinase inhibitors or by the knockdown of CDC2/CDK1 with small interfering RNA and can be promoted by the expression of exogenous CDC2/CDK1 [182]. This is confirmed by other studies in which elevated levels of the CDC2 protein in MCF-7 breast tumor cells were associated with escape from senescence [180]. In subsequent work, Wu's group reported that the aberrant expression of CDK1 promotes the formation of polyploid senescent cells during TIS [183]. Following the promising results of the research on the participation of CDC2/CDK1 in the escape from TIS, it was decided to investigate the downstream target of CDC2/CDK1—survivin. It has been proven that upregulation of survivin causes the escape from senescence and facilitates reentry into the cell cycle [184]. The role of survivin in the escape of cells from the senescence state is confirmed by studies of replicative senescence of melanoma cells, in which, as in the case of H1299 cells, transfection with survivin leads cells to transition from senescence back to the state of proliferation [185]. Interestingly, the reported TIS reversibility studies did not investigate the involvement of signaling pathways involved in escaping from replicative senescence, in particular TP53, p21WAF-1, p16^INK4a^, and RB.

There are also reports suggesting that the cells that escape from senescence are likely cancer stem cells. Such assumptions were suggested based on the expression of ABCG2, which is a marker of cancer stem cells [184]. Cancer stem cells are believed to constitute a small percentage of the neoplastic cell population; however, they are the basis for tumor regrowth and growth of the neoplastic cell population. Another study shows that the recovery of cell subpopulations of TP53 wild-type A549 non-small cell lung cancer cells after the induction of senescence by drugs such as etoposide, m-AMSA, and ICRF-187 is ∼1% [186]. Additionally, a similar number of cells expressed the stem cell markers CD34 and CD117, which prompted the authors to suggest that the escaped cells could be derived from the stem cell population; however, it has not been proven that recovered cells were derived from the senescent cell population.

Other studies also object to the reversibility of senescence and the role of cancer stem cells in this process. A study of irradiated adenovirus-transformed rat embryo fibroblasts showed that a significant proportion of the cell population had replicated DNA while not proliferating, resulting in large polyploid cells that expressed *β*-galactosidase and had morphological features indicative of senescence [184]. Interestingly, >94% of cells remained viable and did not undergo apoptosis, and cells that began to re-proliferate expressed the stem cell markers OCT3/4, Nanog, and SOX2, but this time it was also not proven that they were escaping from senescence. More convincing evidence relating to the recovery of stem cells from senescence is derived from the work of Achuthan et al. [187]. Here, multiple breast tumor cell lines were exposed to various chemotherapeutic drugs accompanied by live-cell imaging of the senescent population. While the bulk of the tumor cell population was eliminated by the chemotherapeutic agents, a small subpopulation of residual surviving senescent cells ultimately gave rise to proliferating cells with stem cell-like properties, in particular based upon the markers CD133 and OCT4.

There is, however, a study confirming the involvement of stem cells in the escape from senescence. In this study, multiple breast cancer cell lines were exposed to various chemotherapy drugs accompanied by live-cell imaging of the senescent population [187]. It was noted that the chemotherapy drugs eliminated most of the cancer cells, but a small subpopulation of the remaining senescent cells survived and began to proliferate to form a new population of cells that exhibited stem cell features, in particular based upon the markers CD133 and OCT4.

## 11. Reprogramming into Stem Cells Compromised by Senescence

The phenomenon of senescence is recognized as one of the factors negatively affecting reprogramming towards induced pluripotent stem cells, as the process of changing cell fate is initiated by transgene delivery, which inevitably involves the induction of cellular stress and the subsequent increase in the expression of genes coding TP53, p16^INK4a^, and p21^CIP^ [188]. In 2016, Mosteiro et al. reported that senescence is relevant for reprogramming of mature cells to iPSc [189]. The increased expression of senescence inductor genes is characteristic for not fully reprogrammed iPSc (“pre-iPSc”) [188]. It was suggested that this phenomenon analogously relates to direct reprogramming and the introduction of even one transcription factor into cells may trigger a cascade of events leading to senescence [120]. It has been documented that senescence (induced by TP53 activation) negatively affects the efficiency of direct conversion of somatic cells to neurons [190]. It has already been proven that TP53 protein plays an important role in the reprogramming process [191–193]. Introduction of transcription factors, including c-MYC or KLF4 oncogenes, initiates a cellular response: TP53 activation and synthesis of p21—a CDK kinase inhibitor that negatively affects cell proliferation [191–193]. In addition, TP53 is a negative regulator of the expression of one of the key genes necessary to obtain stable iPSc lines—Nanog [194]. The senescence process observed in directly reprogrammed cells may therefore suggest an imbalance between TP53 activity and introduced transcription factors. Thus, it was reasonable to use an additional vector coding TP53 with a dominant-negative mutation (dnmTP53) during direct reprogramming of somatic cells to induced neural stem cells [195]. Generally, tumor suppressor genes demonstrate homo- or hemizygous mutations, but TP53 is an exception—in cells with heterozygous mutation of this gene a dominant-negative effect and/or gain of function is observed, which is additionally supported by an unknown mechanism causing higher expression of the mutant allele (compared with the normal allele) [196]. Although the introduction of dnmTP53 into a mixture of transcription factors during direct reprogramming reduces the activity of the normal TP53 present in the reprogrammed cell and positively affects the efficiency of the process itself, this action is only of a research nature, yet it is known that cells containing such a construct are not clinically useful.

Senescence in the context of the reprogramming of somatic cells is a quite new phenomenon, currently gaining more recognition. Not only reprogramming to pluripotent cells but also direct reprogramming into neural stem cells is characterized by low efficiency and proliferation arrest. Direct reprogramming was intended to explore the mechanism of reprogramming, as well as to minimize the risk of teratoma formation, associated with iPSc-based therapy (importantly, iPSc derivatives) [197]. Most of the current scientific reports refer to the direct reprogramming of mouse fibroblasts or experiments in which progenitor cells or iNS-like cells are obtained from human somatic cells [198–200]. The results described in the article by Yu et al. confirmed that the direct generation of induced neural stem cells (iNScs) from somatic cells is limited by senescence [201]. It was also indicated that directly reprogrammed motor neurons, rather than iPSc-derived ones, maintained the senescence hallmarks of old donors, including extensive DNA damage, loss of heterochromatin, nuclear organization, and increased SA-*β*-Gal activity [202]. Direct reprogramming with the use of two transcription factors (SOX2 with c-MYC) certainly increases the reproducibility of this process [120]. Winiecka-Klimek et al. proved that senescence may be one of the reasons for the difficulty of obtaining (iNSc) by direct reprogramming [120]. Since then, other teams have drawn similar conclusions [203]. Despite the fact that SOX2 overexpression influences cell proliferation by regulating oncogenic pathways (including Wnt/*β*-catenin, PI3K/mTOR, JAK/STAT3, and EGFR signaling), it is rather insufficient to accelerate the molecular conversion needed to maintain NSC character. Data also suggest that c-MYC, one of the earliest acting transcription factors, reduces the problem of senescence [204]. It has also been demonstrated that c-MYC inhibits TP53-induced activation of p21 transcription [205]. On the other hand, the same transcription factor, being a proto-oncogene, may contribute to the induction of OIS [206]. It is also known that mouse cells, in particular embryonic mouse cells (mouse embryonic fibroblasts, MEFs), are reprogrammed much more easily and with higher efficiency than human cells obtained from an adult body [198]. Furthermore, mouse cells are less susceptible to replicative senescence [207, 208].

## 12. Cellular Senescence as Difficulty to the Effectiveness of the CAR-T Therapy in Hematological Malignancies

When writing about a dark side of senescence during cancer treatment, we cannot ignore the role of proliferation inhibition in advanced therapy medical products (ATMPs) based on substantially manipulated cells such as CAR-T [31]. Both exhaustion and senescence of T cells are known critical dysfunctional states that impair the effectiveness of the CAR-T therapy.

The *ex vivo* manufacturing process of CAR-T *per se* induces senescence extremely quickly [209, 210]. It was shown within clinical trials that in some leukemia patients CAR-T have achieved spectacular remissions; however, some of the patient treatments failed. Interestingly, the reason for that may be the senescence of the autologous cells. Even more, Aleksandrova et al. showed that cells from healthy donor leukapheresis might be different regarding cell senescence [211]. Zhu et al. showed that an AC133-specific chimeric antigen receptor, developed against AC133 epitope of CD133-positive cancer stem cells, upon contact with patient-derived GB cells, caused rapid upregulation of CD57 on the therapeutic CAR-T, a molecule known to mark near-terminally or terminally differentiated T cells (*i.e.*, senescent) [212].

In general, T-cell exhaustion is one of the most frequently mentioned mechanisms responsible for the failure of CAR-T therapy. The term “exhaustion” is well known in respect to immune system cells and refers to a state of dysfunction characterized by reduced effector function in the form of loss of cytotoxic function and, especially, increased expression of receptors that suppress the immune checkpoints. One of the hallmarks of such “exhausted” lymphocytes is their impaired ability to proliferate upon contact with an antigen [213, 214]. It is especially important to distinguish both terms, the T-cell senescence, which is a quite new and incompletely understood phenomenon, and T-cell exhaustion, since both terms have similar characteristics [209]. It is not clear at the moment whether the exhaustion of lymphocytes results directly from senescence, or whether independent phenomena are observed, even simultaneously, which could be indicated by, e.g., a different expression of specific receptors [215]. T-cell-exhausted phenotype develops when repeated activation of T cells during chronic infection or tumor progression takes place [216] and is considered reversible. The T-cell senescence in the majority of studies is considered irreversible [217]. However, some studies undermine this statement and indicate the role of mitogen-activated protein kinase (MAPK) inhibition in regaining the cell cycle activity and proliferative potential of T cells [218].

T-cell senescence and high expression of inhibitive receptors, PD-1, CTLA-4, TIGIT, LAG-3, CD244, CD160, and TIM3, were observed following T-cell activation during CAR-T preparation [31]. On the other hand, problems regarding the abovementioned receptors may be solved by external pharmacological intervention methods, e.g., the use of fludarabine/cyclophosphamide lymphodepletion regimen or treatment with PD-1 inhibitors [31]. Moreover, the strategy in which the “exhaustion-resistant” CAR-T are generated to prevent, e.g., inhibitory receptor signaling seems to be a more effective approach than modulating their exposure to tumor antigens in tumor microenvironment [213]. Several combination therapies with CAR-T and checkpoint blockade agents, e.g., PD-1 blockade, are currently developed and tested [219–221]. In this respect, the work on the fourth generation of CAR-T and the role of PD-1 and PD-L1 is extremely important. Fourth-generation CAR-T may contain regions encoding immune checkpoint inhibitors (e.g., anti-PD1, anti-CTLA-4, or anti-PD-L1). CAR-T secreting these inhibitors prevents T-cell depletion, reduce tumor growth and mass several times in the case of solid tumors compared with the action of parental CAR-T, and increase the level of secreted granzyme B [222].

## 13. Conclusion/Future Prospect Section

A better understanding of the mechanisms of senescence should lead to an increase in the effectiveness of anticancer therapies. However, it is important to decide whether the senescence of neoplastic cells can be reversible or not. In addition, understanding why senescence occurs rapidly during *in vitro* primary cell cultures can create better conditions for this cell cultivation and thus their comprehensive study. Senescence probably limits the possibility of reprogramming mature cells to multipotent state, so overcoming this obstacle could open the way for an alternative to iPSc technology. Finally, controlling senescence can help to improve the effectiveness of CAR-T therapy.

## Abbreviations

ADCC: Antibody-dependent cellular cytotoxicity
APC: Anaphase-promoting complex
ATMPs: Advanced therapy medicinal products
Bax: B-cell lymphoma protein 2-associated X
BCL2: B-cell lymphoma protein 2
CAR-T: Chimeric antigen receptor T cell
CDK: Cyclin-dependent kinase
CDKI: Cyclin-dependent kinase inhibitors
CNS: Central nervous system
CPC: Cardiac progenitor cell
D + Q: Dasatinib and quercetin
DKD: Diabetic kidney disease
DM: Double minute
dsDNA: Double-stranded DNA
ECASP: Extra centrosome-associated secretory phenotype
EGFR: Epidermal growth factor receptor
EGFRvIII: Epidermal growth factor receptor variant III
GADD45: The growth arrest and DNA damage-inducible 45
GASC: Glioma-associated stromal cell
HR: Homologous recombination
IDH1: Isocitrate dehydrogenase 1
INCENP: Inner centromere protein
iNSc: Induced neural stem cells
IPF: Idiopathic pulmonary fibrosis
iPSc: Induced pluripotent stem cells
IR: Ionizing radiation
MAPK: Mitogen-activated protein kinase
MHC: Major histocompatibility complex
MMP3: Matrix metalloproteinase 3
NF-*κ*B: Nuclear factor kappa-light-chain-enhancer of activated B cells
NK: Natural killer
NSCLC: Non-small cell lung cancer
OIS: Oncogene-induced senescence
PARP1: Poly(ADP-ribose) polymerase 1
PDGFR: Platelet-derived growth factor receptor
PICS: PTEN-induced cellular senescence
ROS: Reactive oxygen species
S/MARs: Scaffold/matrix attachment regions
SAC: Spindle assembly checkpoint
SAHF: Senescence-associated heterochromatin foci
SAS: Senescence-associated secretion
SASP: Senescence-associated secretory phenotype
SA-*β*-Gal: Senescence-associated beta-galactosidase
SCAP: Senescent cell antiapoptotic pathway
SCAPT: Senescent cell antiapoptotic pathway
SIPS: Stress-induced premature senescence
TIS: Therapy-induced senescence
uPAR: Urokinase-type plasminogen activator receptor
VEGF: Vascular endothelial growth factor.
